# YRNAs and YRNA-Derived Fragments as New Players in Cancer Research and Their Potential Role in Diagnostics

**DOI:** 10.3390/ijms21165682

**Published:** 2020-08-08

**Authors:** Kacper Guglas, Iga Kołodziejczak, Tomasz Kolenda, Magda Kopczyńska, Anna Teresiak, Joanna Sobocińska, Renata Bliźniak, Katarzyna Lamperska

**Affiliations:** 1 Laboratory of Cancer Genetics, Greater Poland Cancer Centre, 61-866 Poznań, Poland; kolenda.tomek@gmail.com (T.K.); mg.kopczynska@gmail.com (M.K.); anna.teresiak@wco.pl (A.T.); renata.blizniak@wco.pl (R.B.); 2 Postgraduate School of Molecular Medicine, Medical University of Warsaw, 02-091 Warsaw, Poland; kolodziejczak.iga@gmail.com; 3 Department of Cancer Immunology, Chair of Medical Biotechnology, Poznan University of Medical Sciences, 61-701 Poznań, Poland; a.s.sobocinska@gmail.com; 4 International Institute for Molecular Oncology, 60-203 Poznań, Poland

**Keywords:** YRNA, YRNA-derived fragments, short noncoding RNA, pseudogenes, cancer, biomarker

## Abstract

YRNAs are a type of short, noncoding RNAs. A total of four different transcripts can be distinguished, which are *YRNA1*, *YRNA3*, *YRNA4* and *YRNA5*. All YRNAs are relatively small, made up of about 100 nucleotides each. YRNAs are characterized by a stem-loop structure and each part of that structure carries a different function. YRNAs are transcribed in the nucleus by RNA polymerase III. Then, the YRNA molecule is bound to the polyuridine tail of the La protein responsible for both its nuclear retention and protection from degradation. They also bind to the Ro60 protein, making the molecule more stable. In turn, YRNA-derived small RNAs (YsRNAs) are a class of YRNAs produced in apoptotic cells as a result of YRNA degradation. This process is performed by caspase-3-dependent pathways that form two groups of YsRNAs, with lengths of either approximately 24 or 31 nucleotides. From all four YRNA transcripts, 75 well-described pseudogenes are generated as a result of the mutation. However, available data indicates the formation of up to 1000 pseudogenes. YRNAs and YRNA-derived small RNAs may play a role in carcinogenesis due to their altered expression in cancers and influence on cell proliferation and inflammation. Nevertheless, our knowledge is still limited, and more research is required. The main aim of this review is to describe the current state of knowledge about YRNAs, their function and contribution to carcinogenesis, as well as their potential role in cancer diagnostics. To confirm the promising potential of YRNAs and YRNA-derived fragments as biomarkers, their significant role in several tumor types was taken into consideration.

## 1. Introduction

Most of the human genome (approximately 98%) is built of non-coding RNAs [1,2,3]. Despite the fact that these RNAs do not code proteins, they are essential in gene regulation processes that occur in various ways. Non-coding RNAs are divided into groups depending on the transcript size. Short, non-coding RNAs are smaller than 200 nucleotides and long, noncoding RNAs consist of more than 200 nucleotides [1,2,3]. Long noncoding RNAs function both as tumor suppressors as well as oncogenes and play a crucial role in cancer development and survival by regulating gene expression in processes such as apoptosis, cell proliferation, metastasis, angiogenesis and different types of cellular stresses [4]. Short non-coding RNAs are a class of RNAs that include many different types of transcripts such as microRNA (miRNA) [5], small interfering RNA (siRNA) [6], piwi-interacting RNA (piRNA) [7], small nucleolar RNA (snoRNA) [8], tRNA halves (tiRNA) [9], tRNA-derived fragments (tRFs) [9] and YRNA [10]. Recently, a new class of YRNAs called YRNA-derived small RNAs was also discovered, however information concerning these RNAs is still limited [11,12,13].

In this review, current knowledge about YRNAs and YRNA-derived small RNAs, as well as their function in the carcinogenesis process and potential implication in cancer diagnosis is described.

## 2. Genome Localization and Biogenesis of YRNAs and YRNA-Derived Fragments

YRNAs are highly conserved structures [14], however they are present in all vertebrate species (humans, mice, monkeys) [15]. Interestingly, structures similar to YRNAs are also found in insects (*Bombyx mori*, *Anopheles gambiae*) and prokaryotes (*Mycobacterium smegmatis*, *Deinococcus radiodurans*) [15]. What is more, the number of YRNA transcripts vary between different species [14]. Human YRNA genes are clustered on a single chromosomal locus on chromosome 7q36. YRNAs are relatively small, built of approximately 100 nucleotides each. Four different transcripts of YRNAs can be distinguished: *YRNA1* (112 nt, 35.7 kDa), *YRNA3* (101 nt, 32.2 kDa), *YRNA4* (93 nt, 30.0 kDa) and *YRNA5* (83 nt, 27.6 kDa) [10]. *YRNA2* was originally also distinguished, however later it was discovered to be a degradation product of *YRNA1* and was removed from the list [14,15].

A previous study suggested that YRNAs may undergo similar biogenesis pathways to miRNAs, due to the characteristic stem-loop structure of both molecules [15]. However, YRNAs appear to be transcribed in the nucleus by RNA polymerase III and the YRNA molecule is bound to the polyuridine tail of the La protein (SSB: small RNA-binding exonuclease protection factor [16]) which ensures its nuclear retention and protection from exonucleolytic degradation. YRNAs are also bound to Ro60s (SSA or TROVE2-TROVE domain family member 2 [17,18]), which promote nuclear export and make molecules more stable in the cytoplasm [16]. YRNAs are transported from the nucleus to the cytoplasm using Exportin 5 and Ran GTPase, as is the case with miRNAs [5]. Interestingly, *YRNA3* may enter an alternative transport pathway by way of binding to zipcode-binding protein 1 (ZBP1) and enabling export through Exportin-1 [19]. The intracellular localization of YRNAs depends on three aspects: (i) cell type, (ii) type of Ro60-YRNA complex and (iii) cellular stress. The Ro60 protein is found to be in both the nucleus and cytoplasm, and by binding to YRNAs, protects them from exonucleolytic degradation [14,15,19]. The Ro60 complex with *YRNA5* is localized mainly in the nucleus, but the rest of Ro60-YRNA complexes are found mostly in the cytoplasm [14,19]. It was found that the Ro60 protein and YRNAs also accumulate in the nucleus after exposure to UV irradiation and oxidative stress. Furthermore, YRNAs may also be found in extracellular vesicles as well as in viruses, such as murine leukemia virus (MLV) and human immunodeficiency virus (HIV) [14].

**Figure 1:**
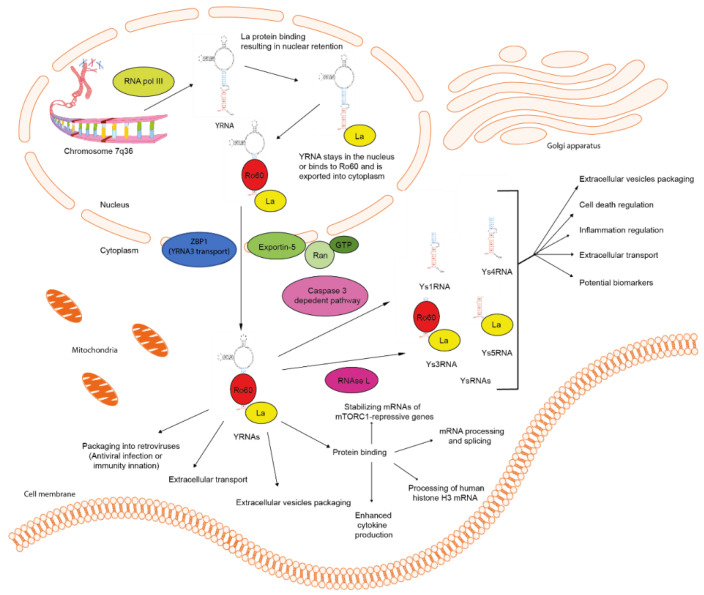
Potential biogenesis pathway of YRNAs and YsRNAs. YRNAs are transcribed from chromosome 7q36 by RNA polymerase III. Next, they are combined with La proteins and Ro60 in the nucleus. Finally, these YRNAs are transported into the cytoplasm with La proteins and Ro60s. There they are distinguished to carry different functions or to be processed further into YRNA-derived fragments [5,13,14,15,16,17,18,19,20,21].

YRNA-derived fragments (YsRNAs) are produced in apoptotic cells due to YRNAs’ degradation performed by caspase-3 dependent pathway. They form two groups of YRNAs, the first with a length of approximately 24 (22–25 nt) and the second with a length of 31 nucleotides (27–36 nt) [20]. YsRNAs may also be formed by enzyme RNAse L in response to UV exposure or by poly I:C-mediated activation of the innate immune system [19]. It was found that smaller fragments derived from *YRNA5* were bound to the Ro60 protein and the bigger fragments derived from *YRNA3* were bound to both Ro60 and La proteins [20]. The binding sites of Ro60 and La proteins are localized in the lower stem domain of YRNA, suggesting that YsRNAs are derived from the lower stem of YRNA transcripts. Such a combination of YsRNAs with Ro60 and La proteins are resistant to nucleolytic degradation processes [15]. Interestingly, YsRNAs derived from *YRNA3* and *YRNA5* are present in solid tumor samples at a similar rate as the cancer-associated miRNA-21. Thus, these fragments were annotated in miRBase as *hsa-miR-1979* (*Y3sRNA*) and *hsa-miR-1975* (*Y5sRNA*). However further sequencing studies revealed that they are not miRNAs and were deleted from miRBase [20]. What is more, these molecules do not enter the miRNA biogenesis pathway, nor do they bind to protein Ago2. They are also independent of Dicer enzymes. It was discovered in the luciferase reporter assay that these fragments do not regulate the gene expression as the RNAi molecules [20]. On the other hand, YsRNAs were found to be important factors in apoptosis and promotion of the inflammation process in monocytes/macrophages [8]. Extracellular YsRNAs induce the caspase-3 dependent cell death and NF-kB dependent inflammation in monocytes/macrophages. Interestingly, intracellular YsRNAs seem to have a similar function, however the promotion of apoptosis and inflammation processes in monocytes/macrophages occurs through activation of Toll-like receptor 7 (TLR7). It is worth noticing that YRNAs do not increase either apoptosis or inflammation, suggesting that TLR7 may be activated only by YRNA-derived fragments [13]. YsRNAs may also be found circulating in serum and plasma by being bound to proteins with a mass of between 100 and 300 kDa, which is believed to stabilize them in bodily fluids [21]. The schematic mechanism of YRNAs and YsRNAs biogenesis is shown in Figure 1.

## 3. Secondary Structure of YRNAs and Function of Domains

**Figure 2:**
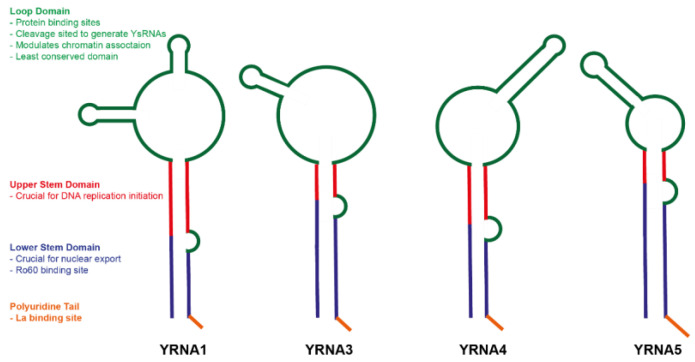
Secondary structures of YRNA1, YRNA3, YRNA4 and YRNA5 with marked functional domains [15].

During biogenesis, YRNAs take the shape of stem-loop structures. YRNAs consist of a loop domain, upper stem, lower stem and a polyuridine tail (Figure 2). Interestingly, the upper and lower stems of YRNAs are highly conserved, however the internal loop varies among specific YRNA transcripts. The least conservative loop domain modulates chromatin association and carries cleavage sites to generate YsRNAs. What is more, it carries a few protein binding sites, such as nucleolin, ZBP1 and polypyrimidine tract-binding protein (PTB). Since the exact effect of binding the aforementioned proteins is still not clearly understood, it is possible that they might modulate the subcellular localization of Ro60 protein and grant specialized cellular functions by binding to specific YRNAs [14,15]. Next, the upper stem domain is essential for the initiation of DNA replication, leading to the formation of new DNA replication forks on human chromosomal DNA. Functional inactivation of YRNAs leads to DNA replication inhibition, arrested development and early embryonic death [14,15]. The lower stem domain is responsible for nuclear export of the YRNAs and carries a Ro60 binding site. The binding of Ro60 to YRNA is dependent on RNA sequence, either specific interactions or shape complementarity. The two bulges in the lower stems of YRNAs deform its helical structure, resulting in the creation of a major groove in the RNA available for binding the amino side chains of the Ro60 protein [15]. Finally, the polyuridine tail is a binding site for an La protein. The La protein is crucial for accurate and efficient termination of RNA polymerase III transcription and binds to the 3′ polyuridine tails of newly synthesized RNAs that are localized in the nucleus. What is more, La proteins are also involved in the nuclear retention of YRNAs and with Ro60 protect YRNAs from exonucleolytic cleavage [14,15].

## 4. Function of YRNAs and YsRNAs

YRNAs are components of ribonucleoproteins (RNPs) Ro60 combined with La proteins. Ro60 uses different types of YRNAs as a scaffolding element [22]. What is more, it’s responsible for intracellular transport of RNA-binding proteins [23], RNA quality control [24,25] and response to environmental stress [14,15]. Additionally, Ro60 binds miss-folded or aberrant non-coding RNAs, such as U2snRNA or 5S rRNA [26,27]. They also form complexes with autoantigens which are targets for the autoimmune system in patients with autoimmune diseases, such as systemic lupus erythematosus (SLE) and Sjogren’s syndrome [14,15]. It was indicated that YRNAs are involved in cellular processes that sustain cell proliferation and targeted degradation of YRNAs inhibits cell proliferation [21,28]. Moreover, they are crucial for initiation of DNA replication, creating new DNA replication forks [15]. Interestingly, YRNA’s accomplish DNA replication independent of Ro60 and La protein [29,30]. YRNAs also carry many protein binding sites, for example those for nucleolin, PTB and ZBP1 and for twenty more different proteins. It is believed that binding specific YRNAs to different kinds of proteins results in determining the subcellular localization of YRNAs and their specialized cellular functions [15,19]. For example, *YRNA3* binds specifically to ZBP1 which prompts an alternative pathway for nuclear transport of *YRNA3* [19]. Moreover, YRNAs can bind with heterogeneous nuclear ribonucleoprotein I (hnRNP I), heterogeneous nuclear ribonucleoprotein K (hnRNP K), Ro RNP-binding protein (RoBPI), Y-box binding protein 1 (YBX1), Y-box binding protein 3 (YBX3), ELAV-like RNA binding protein 1/human antigen R (ELAVL1/HuR), cleavage and polyadenylation specific factor 1 (CPSF1), cleavage and polyadenylation specific factor 2 (CPSF2), factor interacting with PAPOLA and CPSF1 (FIP1L1), symplekin (SYMPK), ELAV-like RNA binding protein 4 (ELAVL4/HuD) and more. Many of the proteins bound to YRNAs function in processing or splicing mRNA transcripts. Some of those bound to *YRNA3* also mediate 3′ end processing of human histone H3 mRNA. Another protein combined with *YRNA3*, HuD, is specifically expressed in neuronal cells where it enhances translation efficiency by stabilizing the mRNAs of mTORC1-repressive genes. The HuR protein, which also interacts with *YRNA3*, can bind to AU-rich elements in mRNA transcripts, resulting in enhanced cytokine production. Other proteins which react with YRNAs, such as MOV10, APOBEC, IFIT5, SYMPK and YBX1 are involved in viral infection or innate immunity. Furthermore, the YBX1 protein plays a role in sorting YRNAs and other small RNAs into extracellular vesicles. Interestingly, 18 out of the 23 proteins that bind with YRNAs were found in extracellular vesicles [19].

**Figure 3:**
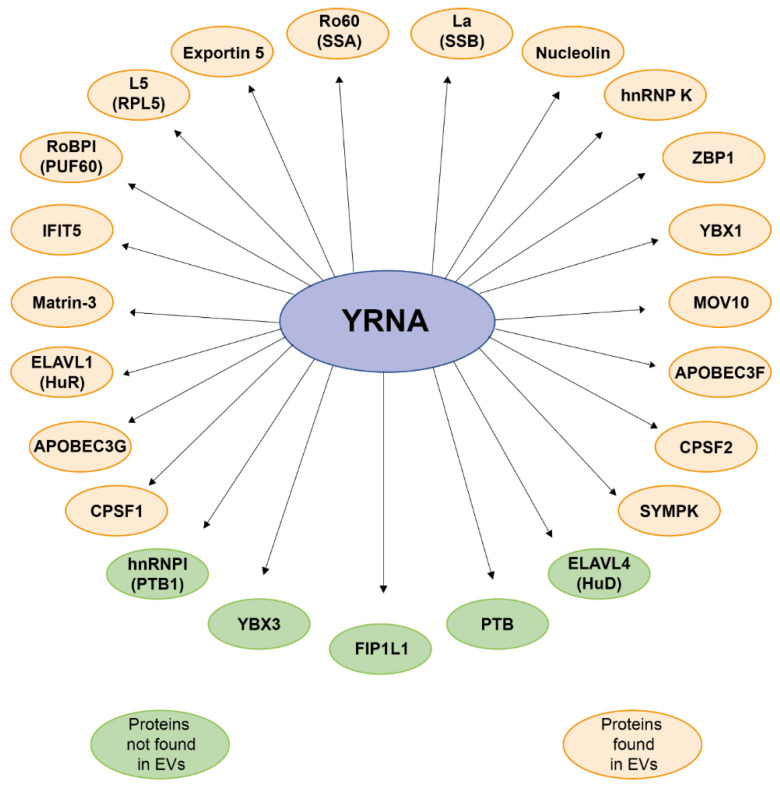
Scheme depicting YRNAs and all proteins bound by all types of YRNAs. Protein name abbreviations are explained in the main text. EV: extracellular vesicles [19].

YRNAs were also found in murine leukemia virus (MLV) and human immunodeficiency virus (HIV). One of the hypotheses explaining the role of YRNAs in viruses is that newly formed YRNAs, but not those bound to Ro60 protein, may act as scaffolds for assembling the virus. On the other hand, YRNAs may also benefit the host of the virus by potentially triggering TLR7 in newly infected cells, resulting in an activation of antiviral immune system response. Finally, it was discovered that YRNAs, as well as many packaged RNAs, can mediate APOBEC packaging, leading in turn to mutations in the genome of the virus and restricting retrotransposition (Figure 3) [19].

YRNA-derived fragments seem to lack gene silencing activity. Unfortunately, the role of YsRNAs is not fully defined yet, but they were found to be dysregulated in many diseases, including cancer [15]. Previous studies showed that YRNA-derived small RNAs regulate cell death and inflammation in macrophages [13]. It was also suggested that YRNAs and YsRNAs might carry a signaling [30] or a gene regulation function [31]. YsRNAs are found to be physiologically relevant in healthy and disease cells. They can be detected in proliferating cancerous and non-cancerous tissues [9]. YsRNAs were found to be significantly dysregulated in breast cancer patients in comparison to healthy controls, and the level of YsRNAs decreased during remission [15]. Furthermore, several studies showed that YsRNAs can be detected in the serum of patients with coronary artery disease (CAD) [11,12]. YsRNAs are also useful in distinguishing patients with Sjogren’s syndrome [21]. What is more, YsRNAs were significantly altered in head and neck squamous cell carcinoma (HNSCC) samples compared to healthy tissues [32]. All these results show that YRNA-derived fragments may be used as biomarkers of different diseases, including cancer.

## 5. YRNA Pseudogenes

Pseudogenes are copies of genes that have lost their function due to an accumulation of different mutations. These mutations may trigger promoters’ inactivation, which are significant frameshifts that cause premature stop codons (or alterations) to appear. Additionally, the translocation of genes to inactive regions of the genome may also take place [33].

Next generation sequencing and bioinformatics methods identified 15,000–18,000 human pseudogenes, and up to 10% of them are transcribed [33,34]. Among them, human YRNA pseudogenes were also identified. According to the NCBI and gene databases, there are 75 YRNA pseudogenes, including 16 *YRNA1* pseudogenes (with a similarity range of 76–91% between parental gene and pseudogenes), 16 *YRNA3* pseudogenes (with a similarity range of 30–96%), 33 *YRNA4* pseudogenes (with a similarity range of 73–97%) and 10 *YRNA5* pseudogenes (with a similarity range of 63–89%). They are all located on different chromosomes, including X chromosome [35,36]. However, available research indicates that there are approximately 1000 human YRNA pseudogenes [30,37].

Due to the fact that pseudogenes may play an important role in diseases including cancer, it is very important to take a closer look at this issue and dispel any doubts or discrepancies. Further studies should focus on whether YRNA pseudogenes possess functions like those of parental genes, or whether they acquire new biological roles or are transcribed without function.

## 6. Cancer-Related YRNAs and YsRNAs and Their Potential as Biomarkers

**Figure 4:**
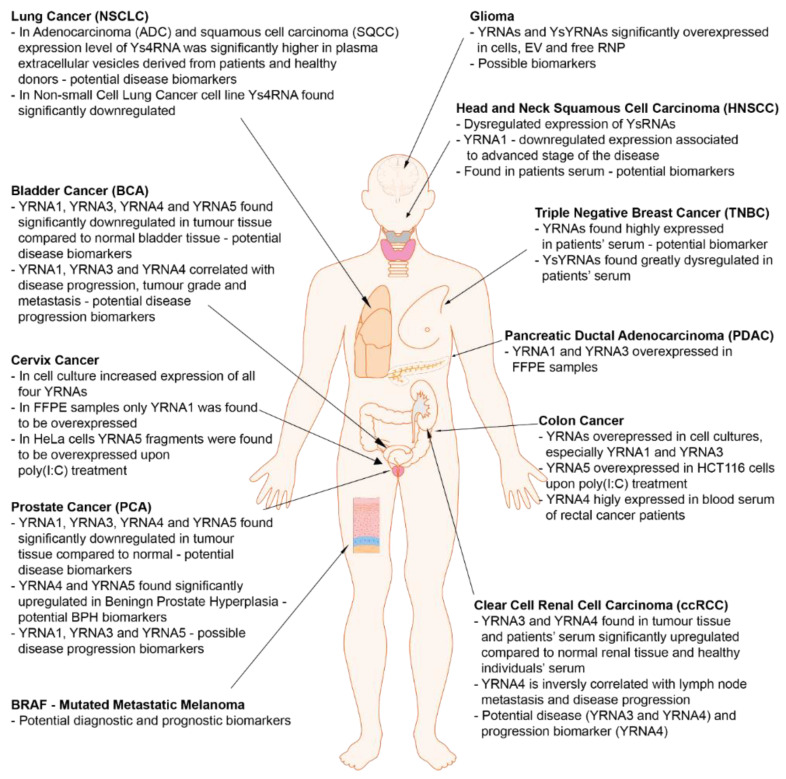
Cancer-related YRNAs and YsRNAs and their potential as biomarkers [1,2,3,20,28,32,38,39,40,41,42,43,44,45,46,47].

Although the exact role of YRNAs has not yet been fully discovered, short non-coding RNAs definitely play an important role in human tumorigenesis. Up till now, the dysregulated expression of YRNAs has been described in several tumor types including bladder cancer, clear cell renal cell carcinoma, prostate cancer, head and neck cancer, triple-negative breast cancer and lung cancer (Figure 4). It is interesting that YRNAs are present not only in tissue but also in serum, blood and plasma and they can be easily extracted from them [1,3,13,14].

The expression profiles of YRNAs in BCA (bladder cancer) and normal urothelial tissue were studied. This comparison revealed that expression levels of all four YRNAs were significantly downregulated and the mean expression levels were 2- to 4-fold lower in BCA than in normal tissue. Interestingly, the expression of *YRNA1*, *YRNA3* and *YRNA4* was highly correlated, while the level of correlation of *YRNA5* to the other YRNA types was significantly less prominent. What is more, Tolkach et al. correlated the expression of YRNAs with clinicopathological features and proved that the expression of three YRNAs (*YRNA1, YRNA3* and *YRNA4*) was strictly associated with advanced stage (lymph node metastasis, muscle invasive BCA) and grade. Furthermore, *YRNA1* and *YRNA3* expression levels were a meaningful predictive indicator for cancer-specific and overall survival among BCA patients, with a clear trend for *YRNA4*. Therefore, in patients with urinary bladder urothelial carcinoma the expression of YRNAs is downregulated and expression changes are related to stage of disease, higher grade and metastasis. This may have prognostic value for overall and cancer-specific survival rates [3]. 

YRNAs also play a significant role in clear cell renal cell carcinoma (ccRCC) and may have prognostic value in this tumor type. Nientdiet et al. indicated increased expression levels of *YRNA3* and *YRNA4* in ccRCC compared to normal renal tissue [2]. Moreover, a decrease of *YRNA4* expressions in patients with lymph node metastasis and in patients with advanced stage of ccRCC was observed. The functional relevance of YRNA4 in pathogenesis was confirmed due to a prominent tendency for poor overall survival following partial or radical nephrectomy in patients with low *YRNA4* tissue levels [2].

In the study of YRNAs, expression levels in prostate cancer (PCA) patients and the possible diagnostic role of YRNAs was investigated. The expression profile of YRNAs was studied in both PCA and normal tissue, as well as in benign prostate hyperplasia (BPH) tissue. The expression levels of all four YRNAs in PCA tissue were 2- to 4-fold lower in comparison to normal prostate tissue. Additionally, in PCA patients, *YRNA4* and *YRNA5* expressions were lower compared to BPH individuals. There was also a correlation between all YRNA expression levels. *YRNA1*, *YRNA3* and *YRNA4* expression was highly correlated, while *YRNA5* level was had a weaker correlation with *YRNA1*, *YRNA3* and *YRNA4*. YRNA levels were also shown to distinguish normal/BPH and PCA tissue. Concerning adverse clinicopathological parameters related to PCA patients, the association of the YRNAs expression with ISUP (The International Society of Urological Pathology) grade group of the tumor was found. Significant differences in the expression levels between ISUP grade groups 1 and 2 were apparent for *YRNA1*, *YRNA3* and *YRNA4*. What is more, the expression of *YRNA5* was interrelated with biochemical recurrence-free survival of prostate adenocarcinoma patients. The study indicates a few possible diagnostic applications of YRNA expression, as well as different biological roles of YRNAs in various tissue and cancer types. In order to confirm the independent prognostic significance of YRNA, further investigations in a larger cohort are still required [1].

The role of YRNA-derived small RNAs was investigated in HNSCC. Martinez et al. revealed the presence of circulating YRNA-derived small RNAs that were remarkably dysregulated in the sera among patients with HNSCC compared to normal tissue samples [32]. The attendance of circulating YRNA-derived small RNAs and the alterations of their expression levels in HNSCC patients may have great potential to serve as noninvasive biomarkers for early disease detection [28]. Moreover, Dhabhi et al. profiled YRNA fragments in serum and tumor tissue from oral squamous cell carcinoma (OSCC) patients. They found that serum levels of 5′ YRNA fragments change in patients with OSCC [3]. Their previous studies indicated a similar trend in patients with breast cancer [38] and patients with HNSCC [39]. The correlation between changes of YRNA-derived fragments’ levels and cancer development may suggest a prospective function associated with tumorigenesis. The contribution of YRNAs and their derivatives in cancer-related processes such as apoptosis, cell proliferation, stress responses and senescence support an association with tumorigenesis [39]. What is more, our unpublished results regarding YRNAs in HNSCC clearly indicate that *YRNA1* expression is downregulated in different HNSCC cell lines, as well as in patients’ tumor samples. However, expression of *YRNA1* was found to be significantly upregulated in stage T4 tumors. It was also observed that higher expressions of *YRNA1* are associated with longer DFS and OS in HNSCC patients. *YRNA1* expression is associated with changes in the expression of many genes involved in carcinogenesis and its high sensitivity and specificity makes it a possible HNSCC biomarker [40].

In a study by Guo et al., several YRNA molecules were identified in the luminal androgen receptor (LAR) subtype of triple-negative breast cancer (TNBC) that may be hormone dependent. Similar to PSA for prostate cancer patients, YRNA fragments detected in the serum of breast cancer patients may provide an opportunity for noninvasive monitoring of the LAR subtype of TNBC in the course of treatment [41].

YRNA-derived small RNAs were also found in non-small cell lung cancer patients (NSCLC). The high-throughput sequencing of small RNAs in plasma extracellular vesicles (EVs) from lung adenocarcinoma (ADC) and squamous cell carcinoma (SQCC) patients and healthy controls was performed, and the analysis revealed that YRNA4-derived fragments were significantly upregulated in plasma EVs among ADC and SQCC patients. Interestingly, YRNA4-derived fragments were downregulated in NSCLC cells and they significantly suppressed the proliferation of NSCLC cell A549. Thus, it all suggests that YRNA4-derived fragments play an important role in NSCLC development and may serve as promising biomarkers for NSCLC diagnosis [42].

Kolenda et al. indicate that the expression of YRNAs could serve a diagnostic utility in the case of BRAF-mutant metastatic melanoma patients. However, there are no significantly different plasma YRNA levels in patients with BRAF-mutated unresectable stage III and IV cutaneous melanoma compared to healthy individuals. In spite of that, the estimation of YRNAs appears to be a prognostic marker for BRAF-mutated metastatic melanoma patients and helps to assess progression free survival and overall survival [43].

In the case of glioma, there is only one study that was dedicated to YRNA expression. The researchers used material derived from cells (patients-derived tumorigenic glioblastoma multiforme cells (GBM)), free RNP and extracellular vesicles including micro-vesicles and exosomes. The control material was obtained from a transcriptome analysis of human and mouse astrocytes, endothelial cells, neurons and microglia. Interestingly, all four YRNAs and YRNA-derived fragments were found to be highly abundant in every examined material. What is more, it was found that in extracellular fractions, there was a significantly higher number of YRNA-derived fragments than YRNAs, especially in RNP, compared to cellular fractions of YRNAs and YsRNAs [44].

Furthermore, increased expression of *YRNA1*, *YRNA3*, *YRNA4* and *YRNA5* were discovered in cervix cancer cell cultures [45]. However, in the FFPE samples derived from cervix cancer patients, only *YRNA1* showed a significant overexpression [46]. It was also indicated that in HeLa cells the expression of *YRNA5* fragments was significantly higher with poly(I:C) treatment [20].

There are also few studies considering YRNAs expression in colon cancer. In colon cancer cell cultures, all four YRNAs were significantly overexpressed, especially *YRNA1* and *YRNA3* [45]. In another study, *YRNA5* was found to be overexpressed in HCT116 cells during poly(I:C) treatment [20]. What is more, in a study based on blood serum derived from rectal cancer patients, *YRNA4* was found to be highly expressed in multiple variants [47].

Finally, in FFPE samples obtained from pancreatic ductal adenocarcinoma (PDAC) patients, *YRNA1* and *YRNA3* were found to be significantly overexpressed [46]. What is more, the expression of Ro60 was also found to be significantly increased in PDAC samples compared to normal tissue samples. The knockdown of Ro60 resulted in a significant decrease in cell proliferation and invasion, making YRNAs a promising possible target for silencing Ro60 in PDAC [48].

An analysis performed using StarBase v2.0 (based on data obtained from The Cancer Genome Atlas, TCGA) showed that YRNAs are dysregulated in many cancer diseases. *YRNA1* expression is changed in seven out of 17 tested cancer types. In two of them the expression of *YRNA1* is upregulated compared to normal samples and in the other five *YRNA1* expression is downregulated. *YRNA4* was found to be dysregulated in four cancer types out of the 17 examined and in all cases the expression of *YRNA4* was downregulated in comparison to normal samples. *YRNA3* was not detected to have changed in any of the 17 examined cancer types, while for *YRNA5* there are no studies available. What is more, neither *YRNA1* nor *YRNA4* showed differences in overall survival in any cancer disease tested [49]. This data is similar to the results previously obtained in YRNAs expression studies described in this paper. However, not all previously described cancer types showed similar results in TCGA patients’ samples, such as bladder cancer or prostate cancer. In the analysis using StarBase v2.0 (which is based on TCGA patients’ samples), previously reported bladder and prostate cancer did not show any significant changes in YRNAs expression. Such differences may occur due to different sample types (tissues, serum, plasma, cell lines) as well as due to the small number of samples in each study described earlier in this article.

## 7. Conclusions and Future Perspectives

Since the discovery of YRNAs in the early 1980s [15], they have been the subject of a number of studies. These studies have focused on YRNAs function in both prokaryotic and eukaryotic organisms, how they exert regulatory influence on gene expression, and how they contribute to diverse physiological and pathological processes. Although this research is still scarce, our knowledge on this subject is continually expanding. Particularly interesting and promising conclusions are made taking into account the presence of YRNAs in cancer and their participation in carcinogenesis. At present, YRNAs have been found in seven types of cancer: BCA, ccRCC, PCA, HNSCC, TNBC, NSCLC, glioma, cervical cancer, colon cancer and PDAC [1,2,3,20,28,32,38,39,40,41,42,43,44,45,46,47,48]. Among these, YRNAs exhibit some features that make them potentially promising diagnostic tools.

As demonstrated in this review, YRNAs and YRNA-derived fragments undeniably show a possible influence on carcinogenesis and have great potential in becoming biomarkers for different types of cancer. Thus, the presence of YRNAs and YRNA fragments as well as changes in their expression can be used in the diagnosis of cancer, especially because they are easy to obtain from blood, serum and plasma. However, it is very important to learn more about their exact role in both healthy cells and cancer cells, which means much more research is needed in this field. The questions that remain unanswered are: (i) how the expression of YRNAs and YRNA-derived fragments is regulated, (ii) how they regulate different types of RNA transcripts and (iii) how interactions among them are dysregulated in cancer. In the future, in vitro and in vivo studies should focus on their biological role and their implication in various important processes, such as proliferation, apoptosis and epithelial-to-mesenchymal transition (EMT) process, as well as the maintenance of cancer initiating cells.
